# Levodopa/carbidopa microtablets in Parkinson’s disease: a study of pharmacokinetics and blinded motor assessment

**DOI:** 10.1007/s00228-017-2196-4

**Published:** 2017-01-18

**Authors:** Marina Senek, Sten-Magnus Aquilonius, Håkan Askmark, Filip Bergquist, Radu Constantinescu, Anders Ericsson, Sara Lycke, Alexander Medvedev, Mevludin Memedi, Fredrik Ohlsson, Jack Spira, Jerker Westin, Dag Nyholm

**Affiliations:** 1 Department of Neuroscience, Neurology, Uppsala University, Uppsala, Sweden; 2 Department of Pharmacology, University of Gothenburg, Gothenburg, Sweden; 3 Department of Clinical Neuroscience, University of Gothenburg, Gothenburg, Sweden; 4 Acreo Swedish ICT, Kista, Sweden; 5 Cenvigo, Uppsala, Sweden; 6 Department of Information Technology, Uppsala University, Uppsala, Sweden; 7 Computer Engineering, Dalarna University, Falun, Sweden; 8 Informatics, School of Business, Örebro University, Örebro, Sweden; 9 Sensidose AB, Sollentuna, Sweden

**Keywords:** Parkinson’s disease, Pharmacokinetics, Pharmacodynamics, Levodopa

## Abstract

**Background:**

Motor function assessments with rating scales in relation to the pharmacokinetics of levodopa may increase the understanding of how to individualize and fine-tune treatments.

**Objectives:**

This study aimed to investigate the pharmacokinetic profiles of levodopa-carbidopa and the motor function following a single-dose microtablet administration in Parkinson’s disease.

**Methods:**

This was a single-center, open-label, single-dose study in 19 patients experiencing motor fluctuations. Patients received 150% of their individual levodopa equivalent morning dose in levodopa-carbidopa microtablets. Blood samples were collected at pre-specified time points. Patients were video recorded and motor function was assessed with six UPDRS part III motor items, dyskinesia score, and the treatment response scale (TRS), rated by three blinded movement disorder specialists.

**Results:**

AUC_0–4/dose_ and *C*_max/dose_ for levodopa was found to be higher in Parkinson’s disease patients compared with healthy subjects from a previous study, (*p* = 0.0008 and *p* = 0.026, respectively). The mean time to maximum improvement in sum of six UPDRS items score was 78 min (±59) (*n* = 16), and the mean time to TRS score maximum effect was 54 min (±51) (*n* = 15). Mean time to onset of dyskinesia was 41 min (±38) (*n* = 13).

**Conclusions:**

In the PD population, following levodopa/carbidopa microtablet administration in fasting state, the C_max_ and AUC_0–4/dose_ were found to be higher compared with results from a previous study in young, healthy subjects. A large between subject variability in response and duration of effect was observed, highlighting the importance of a continuous and individual assessment of motor function in order to optimize treatment effect.

## Introduction

Levodopa is currently the most effective symptomatic treatment for PD, and it remains effective throughout the entire course of the disease. However, as the disease progresses, the therapeutic window narrows and along with the short half-life of levodopa, the symptoms become increasingly difficult to treat [1]. The motor fluctuations that may develop within months to years after levodopa treatment start [2], i.e., off (episodes with Parkinsonism), on (near normal motor function), and on with peak-dose dyskinesia (on with involuntary movement) [3], can in advanced PD patients be associated to the oscillating levodopa plasma concentration [4]. One of the core elements of dose titration and continued treatment is evaluation of patient mobility, and how it changes over time. Today, it largely relies on the patient’s subjective assessment of symptoms and clinicians’ assessment of patient status during the brief moment they meet. An increased understanding of assessments with rating scales in relation to the pharmacokinetics of levodopa may increase the understanding of how to individualize and fine-tune treatments.

Jejunal infusion of levodopa/carbidopa gel, allowing fine-tuning of doses [5] which reduces motor fluctuations in patients with advanced PD [6], has demonstrated the importance of individualized doses and a stable plasma concentration [7]. In theory, oral levodopa administration could have similar effects, up to a certain degree, when administered more frequently, but it is difficult to adhere to [8]. A dose dispenser with alarm and memory function, for dispersible low-dose levodopa microtablets (levodopa [5 mg] and carbidopa [1.25 mg]), has been developed (Flexilev® and MyFid®, Sensidose AB, Sollentuna, Sweden) to facilitate adherence and fine-tuning of oral dosing [9]. The dispenser keeps track of doses and pre-programmed time points for administration and the low-dose microtablets allow a fine-tuned dosing in accordance with patients’ needs [10]. The treatment was used in Sweden on licensed prescription between 2013 and 2014. From June 2016, it was made available for prescription to all patients with advanced PD. Recently, it was also approved (2016) by EMA in 13 further EU countries following the mutual recognition procedure.

The aim was to investigate the pharmacokinetic profiles of levodopa and carbidopa, and to assess motor function following a single-dose microtablet administration in Parkinson’s disease patients.

## Methods

### Study protocol, approvals, registrations, and patient consents

The Uppsala Ethical Review Board approved this study and written informed consent was obtained from all participants. The study was conducted in Sweden in accordance with the ethical principles of the Declaration of Helsinki as adopted by the World Medical Association.

### Inclusion criteria

Patients diagnosed with idiopathic PD at the Uppsala University Hospital’s neurology clinic were screened for eligibility. Eligible patients for this study were currently treated with levodopa and experiencing motor complications, i.e., motor fluctuations, verified with wearing-off questionnaire and/or dyskinesia. Patients were excluded if they had ongoing deep brain stimulation treatment, were pregnant, breast-feeding, had any contraindication for the use of levodopa or carbidopa, or had other reasons for exclusion at the discretion of the investigator.

### Study design and medication

This single center, open-label, single-dose study, involving levodopa/carbidopa microtablets, was conducted at CTC (Clinical Trials Consultants AB) Center at the University Hospital in Uppsala, Sweden, between May and August 2015.

The dose administered was 150% of the calculated levodopa/carbidopa equivalents, a suprathreshold challenge dose as in a previous study [11], to follow the patient’s transition from off-state, to normal mobility and/or evoked dyskinesia, and the regression back. A patient’s dose was calculated from the usual morning dose of levodopa and other anti-PD drugs, because no other anti-PD drug were allowed on the day of the study. On the morning of the study, the patients received a fasting dose of their usual morning dose in levodopa/carbidopa calculated equivalents, after an 8-h washout. The microtablets were dispersed in a glass of water, 100 mL. The patients drank the full volume of dispersed drug while seated.

No other anti-PD drugs were allowed during the pharmacokinetic study day. The patients were allowed to discontinue the further testing prior to the planned protocol stopping time if they could no longer remain without medication.

The levodopa/carbidopa equivalent dose conversion was based on a study and a systematic review [12, 13] comparing the pharmacokinetics and effect between levodopa/carbidopa, levodopa/benserazide, and other anti-Parkinson drug formulations.

The conversion factor was for levodopa/benserazide: milligram of levodopa × 1.2 and for a formulation with entacapone: milligram of levodopa × 1.33. For formulations without levodopa, the conversion factor was for pramipexole: milligram of pramipexole (as salt) × 12.5; for ropinirole: milligram of ropinirole × 2.5; for rasagiline: milligram of rasagiline × 8.33; for apomorphine: milligram of apomorphine × 10; and for amantadine: milligram of amantadine × 1. The prolonged release formulation conversion factors were adjusted for three times daily levodopa administration.

### Pharmacokinetic sampling

Up to 15 samples were collected from each patient, at pre-specified time points; one blood sample prior to dosing, one in conjunction with study dose administration at time 0, and thereafter at 15, 30, 45, 60, 80, 100, 120, 150, 180, 210, 240, 300, and 360 min after dose administration.

### Pharmacodynamic measurements

During the inclusion visit, the patients were asked to answer a part of the Unified Parkinson’s Disease Rating Scale (UPDRS) part IV, questions 32 to 42, and the 9-item wearing-off questionnaire [14].

During the study, the motor function assessment was conducted in repeated test cycles: once before the study dose administration and then repeatedly every 20 min until 111 min, and subsequently every 30 min until 321 min (time of the last test) or until the patient could no longer remain without medication.

The patients were asked to sit on a chair with no armrests. The video recording was started and the patient was instructed to perform the UPDRS-III items in accordance with previous studies [15] and the UPDRS [13]; (1) rapid alternating movements of hands, while seated, one arm at a time, (2) sit, look into the camera, (3) read a text developed by speech therapists for 1 min [16], (4) tap the index finger on the thumb ten times as quickly as possible with the largest amplitude possible, one hand at a time, (5) tap the heel on the ground in rapid succession picking up the entire leg ten times as high and as fast as possible, one heel at a time, and then 6) rise from the chair with arms folded across the chest, let the arms down, walk a few meters, turn, walk back, and sit down.

### Video rating

A computer program was used to randomize the video sequences to ensure that the video recordings were presented in a randomized order to the three movement disorder specialists, here abbreviated as SMA, HA, and RC, to ensure that the rating was blinded with respect to time from dose administration. The six UPDRS items finger tapping (item 23), rapid alternating movements of hands (item 25), tapping the heel (item 26, only rated by two of the raters), arising from chair (item 27), gait (item 29), and bradykinesia (item 31) were rated according to the definitions of the motor examination part of the UPDRS, with a score of 0 to 4, per time point and item. These six items were summed up to a UPDRS item score.

The raters also noted if the patient had choreatic and/or dystonic dyskinesia and rated severity from 0 to 4. The patients overall mobility was also rated, according to the Treatment Response Scale (TRS), a seven step scale ranging from −3 (severe parkinsonism) to 0 (normal mobility) to +3 (severe choreatic dyskinesia) [15]. In the case of mixed patterns, the instructions were to rate according to the dominating movement pattern, with the walking ability weighted as more important.

### Safety assessment

Patients were monitored for adverse events throughout the study.

### Statistical analysis

The pharmacokinetic and statistical analyses were performed with the software R 3.2.2 [17, 18]. The blood samples that were not drawn on the exact time point were approximated to the pre-specified times for the statistical analysis. Each patient was rated regarding six UPDRS items, dyskinesia scores, and TRS score by each of the three raters for all time points. The raters’ median scores for the six UPDRS item scores were summed up per time point into a total value.

The calculated pharmacokinetic parameters were baseline- and dose-adjusted (to 100 mg for levodopa and 25 mg for carbidopa) maximum concentration (*C*_max/dose_) and area under the plasma concentration time curve (AUC_0–4/dose_) of levodopa and carbidopa. The measured concentration at time 0 was subtracted from the rest of the measurements that were then divided with the individual administered dose of each compound and multiplied with 100 (levodopa) or 25 (carbidopa). Patients that remained without additional medication for at least 4 h were included in the analysis of AUC_0–4/dose_. At least three descending measurements after the peak were required for the calculation of *t*_½_. All measurements available were included in the calculation of *t*_½_. The mean time to maximum concentration (*T*_max_) was calculated for all patients. Levodopa and carbidopa AUC_0–4/dose_, *C*_max/dose_, and *t*_½_ were statistically compared with previously reported values in healthy subjects [12]. The statistical analysis of AUC_0–4/dose_, *C*_max/dose_, and *t*_½_ was conducted with the Welch two-sample *t* test, due to unequal variances and number of individuals. The statistical comparison of *T*_max_ was carried out with a Wilcoxon rank sum test.

### Bioanalysis of analytes

The blood samples were collected in EDTA tubes, stored on ice, and centrifuged (20 min, Sorvall SL50T, 3900 rpm) within 1 h. The plasma was stored frozen at −80 °C until analysis (within 6 months). All samples were analyzed at The Department of Pharmacology, University of Gothenburg, Sweden.

After thawing, 0.5 mL of plasma was mixed with 4-dihydroxybenzylamine (1 mg/ml) and added to 500 μL trichloroacetic acid to precipitate proteins. After vortex for 5 min, the samples were centrifuged at 6500 rpm for 10 min at +4 degrees, 5000 RCF. Fifty microliters of supernatant was transferred to a HPLC vial and 3–5 μl was injected on HPLC-ED system.

The HPLC system consisted of a pump (Dionex Ultimate 3000 pump) equipped with a C_18_ reverse phase, 2.0 mm × 200 mm column (Onyx). The system was connected to an auto sampler equipped with a tray cooling kept at +4 °C. The detection device was an amperometric detector (Waters 450). The mobile phase consisted of 50 mmol/L phosphate buffer, pH 2.88 with EDTA 100 mg/L, methanol 4.0%, acetonitrile 1.5%, and 1-octanesulphonic acid 100 mg/L. The method was validated in agreement with the ICH Validation of Analytical Procedures Q2, R1, step 4 version (ICH = International Conference on Harmonization of technical requirements for registration of pharmaceuticals for human use).

Plasma samples were assayed for levels of levodopa and carbidopa. The correlation coefficient (*r*) for linearity determined by the method of least squares was more than 0.995 and the limits of quantification (LOQ) were 10 and 20 ng/mL for levodopa and carbidopa, respectively.

## Results

### Study population

Nineteen patients, 14 male and 5 female, experiencing wearing-off symptoms and/or dyskinesia, were enrolled (Table 1).

**Table 1:** Patient characteristics, *n* = 19

ID	Sex	Age	BMI	Symptom onset (years)	Diagnosis (years)	Start LD (years)	Hoehn & Yahr	UPDRS IV	Wearing off^b, c^ (yes/no)	Dyskinesia^b, d^ (yes/no)	Study dose^e^ LD/CD (mg)	Last blood sample	Last motor function test
A^a^	Male	69	22.8	11	10	10	4	8	Yes	Yes	300/75	300	291
B	Female	70	22.4	11	10	10	4	11	Yes	Yes	220/55	300	321
C	Male	64	26.5	10	6	6	3	14	Yes	Yes	345/86.25	180	171
D ^a^	Male	66	25.5	17	15	14	3	4	Yes	Yes	410/102. 5	240	261
E ^a^	Male	61	22.3	13	11	11	3	5	Yes	Yes	360/90	240	261
F ^a^	Female	82	21.5	12	9	9	3	7	Yes	Yes	360/90	240	261
G	Female	73	25.6	17	15	13	3	9	Yes	Yes	155/38.75	210	201
H ^a^	Male	79	27.7	6	4	4	3	4	Yes	Yes	370/92.5	240	261
I ^a^	Female	76	24.2	23	12	12	3	7	Yes	Yes	250/62.5	300	321
J ^a^	Male	61	24.5	7	4	4	2	3	Yes	No	270/67.5	300	321
K ^a^	Male	80	24.7	7	5	5	2	2	No	Yes	360/90	360	321
L ^a^	Male	74	23.4	8	8	8	4	4	Yes	No	110/27.5	360	321
M ^a^	Male	74	30.0	6	5	5	3	2	Yes	No	250/62.5	300	321
N	Male	80	22.5	35	33	33	5	9	Yes	Yes	250/62.5	180	171
O ^a^	Male	73	22.2	7	6	6	2	5	Yes	No	180/45	360	321
P ^a^	Male	68	28.3	9	9	9	3	9	Yes	Yes	295/73.75	360	321
Q	Male	69	20.0	17	13	13	5	7	Yes	Yes	365/91.25	300	231
R ^a^	Female	65	26.3	4	2	2	3	3	Yes	No	180/45	300	321
S ^a^	Male	72	28.7	12	7	7	2	5	Yes	No	195/48.75	360	321
MEAN	14/5	71.4	24.7	12.2	9.7	9.5	–	–	–	–	275/68.75	285.8	280.0
SD	–	6.3	2.7	7.3	6.8	6.5	–	–	–	–	86.3/21.6	58.7	51.7

*LD* levodopa, *CD* carbidopa

^a^Included in the PK analyses

^b^Reported at inclusion

^c^Based on wearing-off questionnaire

^d^Based on UPDRS IV

^e^Levodopa/carbidopa equivalents based on individual morning dose

The converted anti-PD drugs were levodopa/benserazide (*n* = 15), levodopa/carbidopa (*n* = 4) immediate release, entacapone (*n* = 3), pramipexole (*n* = 4, one with prolonged release formulation), ropinirole prolonged release (*n* = 3), ropinirole immediate release (*n* = 1), rasagiline (*n* = 4), apomorphine (*n* = 2), and amantadine (*n* = 1).

Ten patients remained without additional medication until the last motor function test (321 min), and five patients did not take any additional medication containing levodopa until the last blood sample, at 360 min, was drawn.

### Pharmacokinetics of levodopa and carbidopa

**Fig. 1:**
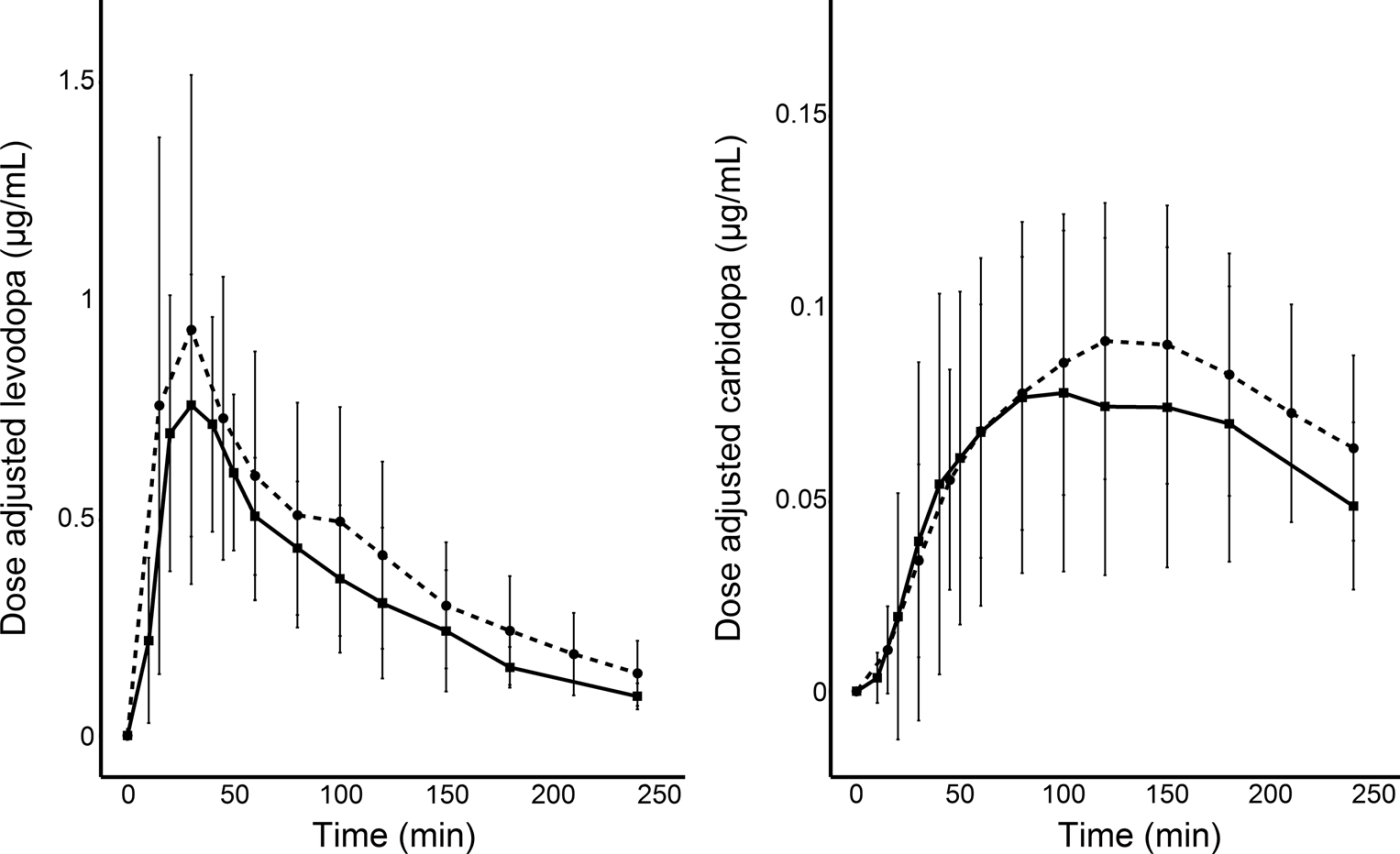
Baseline- and dose-adjusted levodopa (to 100 mg) and carbidopa (to 25 mg) plasma concentration profiles (mean ± SD) for patients over time for patients and non-adjusted for healthy subjects [12]. Pharmacokinetic mean (±SD) profiles of levodopa and carbidopa in plasma (μg/mL), Patients profiles are baseline- and dose-adjusted to 100 mg levodopa and 25 mg carbidopa over time. *Filled triangles*: Patients (levodopa, *n* = 14; carbidopa, *n* = 13), *empty circles*: healthy subjects (*n* = 18). Data from healthy subjects reused with permission from the Wolters Kluwer Health, Inc. (Clinical Neuropharmacology)

All patients had some detectible levodopa at time 0; therefore, baseline- and dose-adjusted *C*_max_ (*C*_max/dose_) and AUC (AUC_0–4/dose_) were calculated (Table 2). One patient had two blood samples drawn 5 and 6 min later than pre-specified time. The time points were approximated to the pre-specified time points. Mean baseline-adjusted *C*_max/dose_ and AUC_0–4/dose_ for levodopa was higher in patients compared with healthy levodopa-naive subjects from a previous study [12] (*p* = 0.026, [0.270, 95% CI: 0.0350–0.505] and *p* = 0.0008, [0.378, 95% CI: 0.179–0.576], respectively) (Fig. 1). *T*_max_ for levodopa and carbidopa did not differ between the patients and the healthy volunteers; however, the times of blood-sampling slightly differed between the comparative study and this study during the first hour. Sampling time points were same in both studies between hour 1 and 3. Carbidopa half-life for patient was found to be longer compared to healthy subjects (*p* = 0.029, [46.0, 95% CI: 5.23–86.7]).

**Table 2:** Pharmacokinetic parameters of levodopa and carbidopa

	Levodopa						Carbidopa					
	N	Mean	SD	Min	Median	Max	N	Mean	SD	Min	Median	Max
Patients												
*C*_max/dose_ (μg/mL)^a^	19	1.17^††^	0.43	0.31	1.16	1.96	19	0.09^†^	0.03	0.03	0.09	0.14
*T*_max_ (min)	19	32	23	15	30^†^	100	19	134	47	80	120^†^	240
AUC_0–4h/dose_^a, b^ (min^c^μg/mL/mg)	14	1.15^††^	0.31	0.38	1.21	1.74	14	0.67^†^	0.26	0.23	0.68	1.14
*t*_½_^d^ (min)	14	106^†^	16	85	104	144	13^e^	171^††^	37	117	173	248
Healthy volunteers [11]												
*C*_max_ (μg/mL)	18	0.90	0.25	0.51	0.88	1.38	18	0.09	0.05	0.03	0.09	0.21
*T*_max_ (min)	18	37	23	20	35	120	18	109	48	40	100	180
AUC_0–4h/dose_^b^ (min^c^μg/mL/mg)	18	0.77	0.17	0.53	0.75	1.13	18	0.58	0.32	0.17	0.52	1.44
*t*_½_^c^ (min)	18	91	34	45	85	198	18	125	72	56	101	315

^a^Baseline and dose adjusted

^b^Time points 0–240 min (0–4 h). Five patients were excluded because they did not have data until 240 min

^c^Reused with permission from the Wolters Kluwer Health, Inc. (Clinical Neuropharmacology)

^d^At least three descending concentration time points were used for the calculation of *t*½. All time points included

^e^Patient F did not have descending time points at end of trial

^†^not found to be significant, compared with healthy volunteers from previous study [12]

^††^ *p* < 0.05, compared with healthy volunteers from previous study [12]

### Pharmacodynamics represented by six UPDRS items, dyskinesia, and TRS scores

**Fig. 2:**
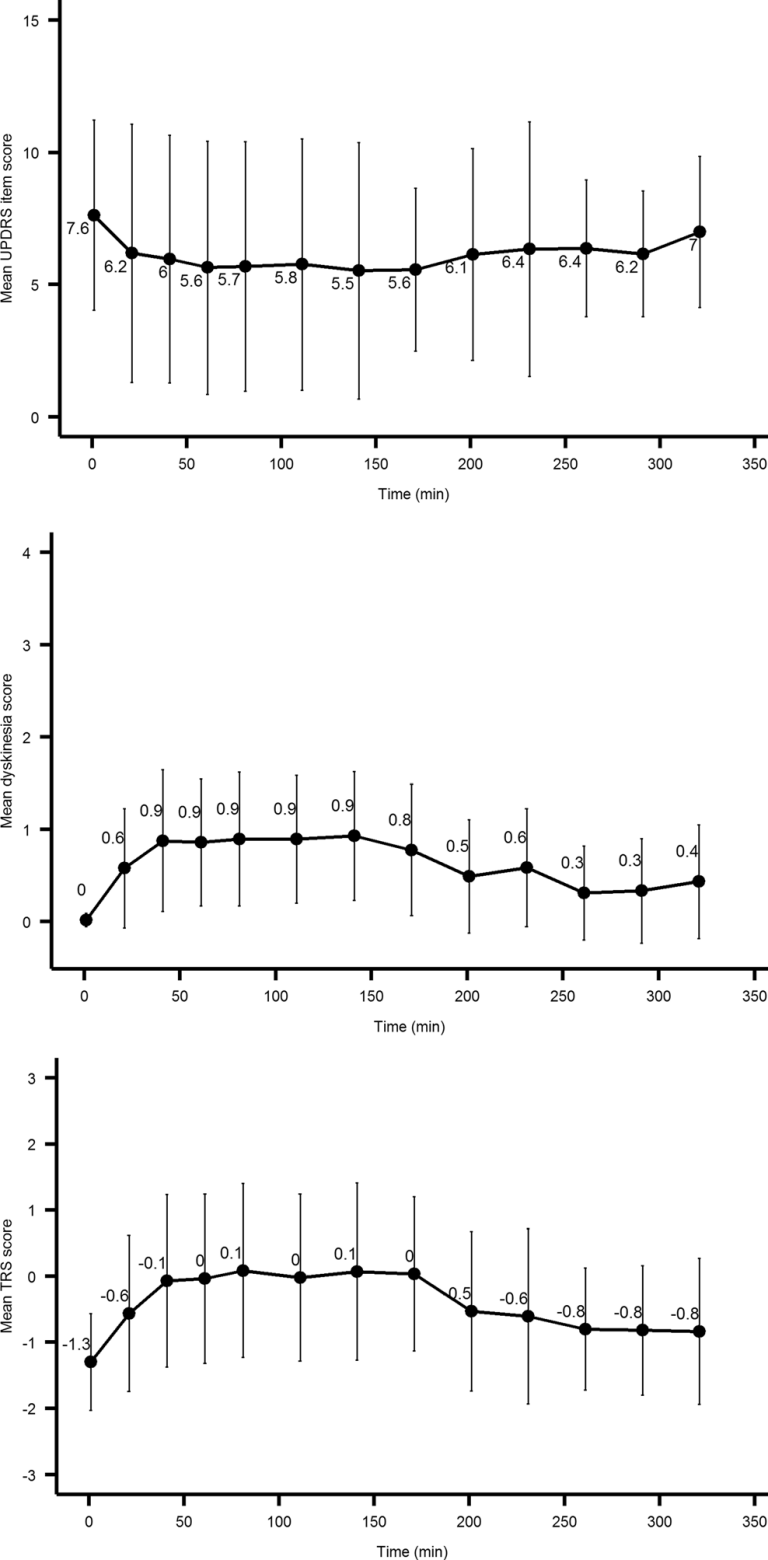
Mean (±SD) UPDRS item score, dyskinesia, and TRS scores (*n* = 19). Motor function assessed over time, after intake of a suprathreshold individual dose of levodopa/carbidopa microtablets

The mean (±SD) sum of the six UPDRS item score, dyskinesia, and TRS scores per time point are shown in Fig. 2. The mean UPDRS item score at dose intake was 7.4, and the mean TRS score was −1.4. The dyskinesia score was 0 for all patients at time 0. Individual results are presented in Table 3.

**Table 3:** Time points (minutes) for improvement and deterioration according to blinded ratings of UPDRS item score, dyskinesia and TRS score for each patient

ID	UPDRS III improvement of ≥2^a^ points	UPDRS III return to baseline^a^	TRS score ≥ 0	TRS score < 0	Choreatic dyskinesia score ≥ 1	Choreatic dyskinesia score < 1
A	41	201	41	201	41	201
B	21	171	21	171	21	171
C	21	–	21	–	21	–
D	21	261	21	261	21	261
E	21	171	21	171	21	171
F	21	201	21	261	21	261
G	41	111	21	111	–	–
H	81	171	–	–	–	–
I	–	–	21	–	111	–
J	201	291	–	–	141	291
K	–	–	–	–	21	–
L	61	81	–	–	–	–
M	141	261	–	–	–	–
N	–	–	–	–	21	–
O	21	201	–	–	–	–
P	21	291	41	291	21	291
Q	41	201	41	201	41	201
R	21^b^	291^b^	21	–	41	261
S	–	–	–	–	–	–
Median^c^	21	201	21	201	21	261
n	15	14	11	8	13	9

UPDRS part III; six items rated from 0 to 4; choreatic dyskinesia, rated from 0 to 4

*TRS* treatment response scale rating from −3 to +3

^a^At most 1.5 points improvement from baseline or worsening (for patients who had an improvement of ≥2 points)

^b^Within this range there were two occasions of temporary improvement/deterioration from baseline

^c^For patients that improved and deteriorated according to the cut off values

The mean (±SD) time to maximum sum of UPDRS item improvement was 79 min(±60) (*n* = 16), excluding the three patients who did not show any improvement, or worsened. *C*_max/dose_ was 1.14 μg/mL (±0.46) and *T*_max_ was 34 min (±24) for the 16 patients. The mean duration of effect was 154 min (±73) (*n* = 14).

The median cutoff value for onset of choreatic dyskinesia was set to 1, meaning that at least two of the raters rated the patient as dyskinetic with at least a score of 1. Six patients did not show any sign of dyskinesia throughout the study (Table 3, Fig. 2). Mean time to onset of dyskinesia was 42 min (±39) (*n* = 13). The mean time to maximum dyskinesia score was 56 min (±37) (*n* = 13).

The mean time to TRS score effect maximum was 54 min (±52) (*n* = 15), excluding patients who did not show any improvement, or worsened. *C*_max/dose_ was 1.15 μg/mL (±0.47) and *T*_max_ was 35 min (±24) for the 15 patients. The mean duration of effect, calculated for the patients that returned to a score of less than 0 on the TRS, was 180 min (±53) (*n* = 8).

### Adverse events

One patient vomited 25 min after dose administration and one patient felt nauseous after 40 min. Two patients fell once during the walking test, at 40 min, and at 60 min after dose administration. There were no injuries and the patients did not discontinue the study. No serious or severe AEs were reported during the study or led to discontinuation or change in therapy.

## Discussion

This is the first pharmacokinetic study where PD patients received levodopa/carbidopa microtablets. The aim was to characterize the pharmacokinetics of levodopa/carbidopa microtablets and to investigate the motor response, during the transition from off-state, to normal mobility and/or dyskinesia and the regression back, in patients with advanced PD following a 50% increased levodopa equivalent morning dose. Since the main inclusion criteria were wearing off symptoms and/or dyskinesia, the group became heterogeneous regarding years since symptom onset, diagnosis, and start of levodopa treatment. Due to PD severity, not all patients could remain without additional medication for 6 h from study start. The individual morning doses administered were in some patients very high, ranging from 110/27.5 to 410/102.5 mg of levodopa and carbidopa, respectively. The range demonstrates the large inter-individual variability in dose requirement that exists among patients [19]. Doses administered were 50% higher than usual, and the equivalence algorithm, which is an approximation to the levodopa equivalent doses, may have exaggerated the doses further causing more dyskinesia than would be observed with the patients optimal doses. Four patients had rasagiline, a MAO-B inhibitor. The study design with only an overnight washout means that the rasagiline effect was not washed out [20]. This could have influenced the motor function by perhaps increasing the peak dose effect and delaying the time to wearing off in the patients.

The pharmacokinetics for the levodopa/carbidopa microtablets in patients were compared to previous reports in healthy subjects [12]. The baseline-adjusted levodopa AUC_0–4/dose_ and *C*_max/dose_ was found to be higher in patients compared with healthy volunteers, an effect that has been previously seen and suggested to be related to long-term levodopa therapy and age [21–24]. The baseline and dose adjustments may however have contributed to a bias, resulting in an underestimation of the AUC_0–4/dose_ and *C*_max/dose_. The patients that were included in the calculation of half-life had measurements for at least 4 h. The time may not be long enough for calculation of carbidopa half-life. Carbidopa half-life was found to be longer in patients compared with healthy subjects.

The complexity behind motor response to different levodopa doses and the individual variability is well known [3]. This can also be observed in the present study. The knowledge of each patient’s individual kinetic-dynamic approach is important for an optimal treatment. It is also clear that frequent assessments are needed to find an optimal dosing. From a practical point of view, objective assessment tools are required, in order to optimize the treatments. The large variation in the results makes it hard to draw strong, general, conclusions about the relationship between the levodopa plasma concentration and the effect, and a model-based approach for the analysis of the data would be appropriate. With the flexibility that the microtablets provide, the individualization of treatment may become easier, with respect to fine-tuned dosing. Further analysis of the results from this study may give individualized guidance on how to determine what the optimal dose is for a patient based on their response to a test dose.

In summary, the systemic dose-adjusted exposure and maximum concentration of levodopa, in a PD population following administration of levodopa/carbidopa microtablets in fasting state, was found to be higher in patients compared with previously reported values from young healthy volunteers. A high between subject variability with respect to response and duration of effect was observed, highlighting the importance of a continuous and individual assessment of motor function in order to optimize treatment effect.
